# Tympanoplasty Type-I using tragal perichondrium graft: Our experience

**DOI:** 10.12669/pjms.35.4.421

**Published:** 2019

**Authors:** Fazal I Wahid, Sajid Rashid Nagra

**Affiliations:** 1 Dr. Fazal I Wahid, FCPS. Department of E.N.T, Head & Neck Surgery, Medical Teaching Institute (MTI), Lady Reading Hospital (LRH) Peshawar, Khyber Pakhtunkhwa, Pakistan; 2 Dr. Sajid Rashid Nagra, FCPS. Department of E.N.T, Rai Medical College Sargodha, Punjab, Pakistan

**Keywords:** Tympanoplasty, Tympanic membrane perforation, Graft. Tragal Perichondrium

## Abstract

**Objective:**

To determine the efficacy of tragal perichondrium graft used in tympanoplasty Type-I at a tertiary care hospital.

**Methods:**

This descriptive case-series study was performed at the department of E.N.T, Head and Neck Surgery, Medical Teaching Institution/Lady Reading Hospital (MTI/LRH), Peshawar, Pakistan from June 2017 to May 2018. After approved from IREB, a well informed consent was taken. Pure Tone Audiometry (PTA) was performed before surgery and post-operatively at three and six months interval. The mean ± SD Air-Bone Gap (ABG) was calculated in pre- and postoperative PTA. The data were analyzed using SPSS (version 20). Chi-square (X2) test of significance was used taking confidence interval at 95%. The p-value ≤0.05 was considered significant.

**Results:**

Total patients were 36; male 21 (58.3%), female 15(41.7%) with male: female ratio of 1.4:1. Mean ± SD age was 27.14 ± 7.49 years (Range 15 – 50Years). Tympanic membrane perforation was commonly found on right side 22 (61.1%), predominantly involving anterio-inferior site 19 (52.8%) and medium sized perforation outnumbered 22 (61.1%). Mean pre-operative air-conduction of 49.72 dB was significantly reduced to 18.27 dB with p-value of <0.05. Similarly the pre-operative mean air bone gap on PTA of 45.63 ± 8.35dB was also reduced to statistically significant level of 7.41 ± 3.51 dB on post-operative PTA with p-value of <.05. Graft was taken up well in 34 cases (94.4%).

**Conclusion:**

Tragal perichondrial graft is an effective grafting material used for tympanoplasty due to its possessing qualities.

## INTRODUCTION

Chronic Otitis Media (COM) is as ancient human problem as humanity itself.^1^ It is the most common treatable cause of hearing loss in developing countries like Pakistan.^2^ In the past the subject of hearing restoration was not properly addressed by otologist, however with the discovery of antibiotics, availability of sophisticated operating microscopes and anesthetic facilities a dramatic revolution has taken place.^3^ Marcus Banzer was the first surgeon to treat the COM with graft obtained from pig’s bladder in 1640. After that various graft material have been tried by numerous surgeons in different times across the world.^4^ Tympanoplasty is the surgery of reconstruction of Tympanic Membrane (TM) perforation and ossicular chain after clearance of disease from middle ear cleft. Tympanoplasty was introduced by Wullstien in 1952 and Zoellner in 1955. The different graft materials used for tympanoplasty are temporalis fascia, cartilage, perichondrium, vein, fat, dura mater, and periosteum etc.^3^,^5^ Temporalis fascia is the most preferable graft material used by surgeon with success rate of 93–97% in primary tympanoplasties. Its advantages are that it can be harvested through same post aural or endaural incision of tympanoplasty, available in sufficient amount, its basal metabolic rate and thickness have great resemblance with TM.^5^ However the drawbacks of fascia graft are high failure rate in atelectatic drum, retraction pocket, subtotal or total perforation and malfunctioning eustachian tube. The postoperative dimension of fascia graft is unpredictable due to its irregularly arranged elastic and fibrous tissues.^5^ In the literature different techniques of cartilage tympanoplasty have been described, like palisade technique, perichondrium / cartilage island, cartilage shield technique and inlay butterfly graft technique.^5^ The success rate of cartilage tympanoplasty is more than 98%.^6^ The concept of cartilage and perichondrium was introduced by Goodhill on the basis of its advantages of low rejection rate, sufficient and easy availability, best tensile strength, possessing conductive qualities similar to TM.^7^ Moreover cartilage metabolism is slow and its nutrition is via diffusion, stable in poor condition like negative pressure and eustachian tube dysfunction etc.^8^ On the other hand the demerits of cartilage graft are that it mechanically reduces the vibratory pattern of TM, participating in impairment of functional results specifically of high tones and producing an opaque tympanic membrane that may hide underlying residual cholesteatoma.^5^ The common factors responsible for failure of tympanoplasty are atelactasis, eustachian tube dysfunction, tympanosclerosis, active suppuration, large perforation, unfavorable middle ear mucosa and revision myringoplasty.^9^ The mechanism of TM healing is described as such that there is prior ingrowths of connective tissue edges over which epithelium migrates till gap of perforation is closed. Considering this physiological principle connective tissue grafts, which are grafts of mesodermal origin like fascia, vein and perichondrium are superior to all other graft materials. Thus connective tissues replace the fibrous component of TM allowing squamous epithelium above and mucosal tissue below the fibrous layer to close the perforation.^10^

The rationale of this study is that tragal perichondrium graft is strong, thin, easily available and easier to manipulate than tragal cartilage. It is not tough like cartilage and has no spring action thus can easily be used to cover the entire TM perforation.

Keeping in view the above properties of perichondrium, this study was conceptualized to look for the success rate of this graft material in our hospital, because such study has never been conducted in our tertiary care hospital before. We planned this study to determine the success rate and surgical outcome of this graft to establish our ward protocol.

## METHODS

This descriptive case-series study was performed at the department of E.N.T, Head and Neck Surgery, Medical Teaching Institute (MTI), Lady Reading Hospital (LRH), Peshawar, Pakistan from June 2017 to May 2018. Sample size was 36, by online.(OpenEpi: Sample Size for X-Sectional, Cohort, and Clinical Trials, taking confidence interval of 95% and the margin of error of 5%. Convenient (Non-probability) sampling technique was exercised. After getting approval from Hospital Ethical Board, an informed consent was taken from all the patients explaining them the procedure, and its outcomes.

### Inclusion Criteria

- All patients of both genders
- Patients in the age range 15 – 50 years.
- Mucosal COM without discharge for at least three months.
- Hearing loss with minimum Air-Bone Gap (ABG) of 15 dB on PTA.

### Exclusion Criteria

- Retraction pocket or middle ear disease with cholesteatoma.
- Dysfunctional ossicular chain and associated otogenic complication.
- Active mucosal COM.
- COM associated with sensori-neural hearing loss

Every patient was evaluated in terms of history, examination and relevant investigations. Ear was examined for side, size, site, margins of perforation, ear drum remnants, middle ear mucosal status and aural discharge. Pure Tone Audiometry (PTA) was performed before surgery and post-operatively at three and six months interval. PTA was performed by same senior audiometrician using Italy made standard two channel clinical audiometer Amplaid 455. Pre- and postoperative thresholds at 0.5, 1, 2, 4 kHz were recorded on PTA. The Air-Bone Gap (ABG) was calculated in pre- and postoperative PTA. Success of tympanoplasty was determined by graft take, closure in AB-Gap and hearing improvement. Every patient was followed for at least six months. The data were collected on a preformed proforma, which was analyzed using SPSS (version 20). Mean ± standard deviation (SD) and frequency + percentage were calculated for quantitative and qualitative data respectively. Chi-square (X2) test of significance was used for comparison of two qualitative parameters. The confidence interval was considered at 95% and the margin of error at 5%. The p-value ≤0.05 was considered significant.

Tympanoplasty was performed by the author keeping in view the well-established principles of otologic surgery. After endotracheal intubation patient was put in supine position, the ear of surgery was toward the surgeon in appropriate position. Adequate aseptic cleaning and draping was ensured. The site of incision was infiltrated with local anesthesia of 2% lidocaine mixed with 1:100,000 adrenaline. A linear cartilage deep incision was made on medial aspect of tragus, sparing approximately two mm of cartilage dome to maintain its cosmesis. Then perichondrium on lateral side of the in-situ tragal cartilage was harvested with help of freer elevator. Then an end-aural approach to the middle was made and self retaining mastoid retractor was applied to view the perforation properly. The margins of perforation were freshened and tympanomeatal flap approximately 5 mm lateral to perforation margins was elevated from 3 to 9 O’clock position. After tucking tympanomeatal flap superiorly, middle ear was properly examined and disease was cleared. Then few pieces of gelfoam were put in middle ear to create bed for perichondrial graft. After placing the graft over the bed, the graft was easily spread medial to the remnants of TM such that no space was left between TM and canal wall. After repositioning the tympanomeatal flap gelfoam pieces were placed over the graft to avoid lateralization. Polyfax impregnated aural pack was put in the ear canal. Patient was put on injectable antibiotics and oral analgesics and antihistamine. Aural pack was removed after ten days. Every patient was evaluated in terms of PTA, Oto-endoscopic examination of TM and hearing improvement on 3^rd^ and 6^th^ months follow up visits.

## RESULTS

This study included 36 patients; male 21 (58.3%), female 15(41.7%) with male: female ratio of 1.4:1. Patients were in age range 15 – 50 years, with mean ± SD age of 27.14 ± 7.49 years. Patients presented in 3^rd^ and 4^th^ decade of life commonly i.e. 15 (41.7%) and 13 (36.1%) respectively. Demographic characteristics of patients were such that tympanic membrane perforation was commonly found on right side 22 (61.1%), predominantly involving anterio-inferior site 19 (52.8%) and medium sized perforation outnumbered N.22 (61.1%) (Table-I). Mean and Standard deviation of average thresholds at 0.5, 1, 2, 4 kHz on PTA were calculated. Chi-square (X2) test of significance was applied on these PTA findings. There was no change in the pre- and postoperative Bone Conduction on PTA i.e. Perichondrial graft had no effect on bone conduction, however the mean pre-operative air-conduction of 49.72 dB was significantly reduced to 18.27 dB with p-value of <.05. Similarly the pre- operative mean air bone gap on PTA of 45.63 ± 8.35dB was also reduced to statistically significant level of 7.41 ± 3.51 dB on post- operative PTA with p-value of <.05. (Table-II). There was significant improvement in patients hearing after tympanoplasty in 28 cases (77.8%) while mild degree hearing improvement and even no hearing improvement was observed in 4 cases (11.1%) and 3 cases (8.3%) respectively. Graft was taken in 34 cases (94.4%) Table-III.

**Table I:** Demographic data including age, gender, side, site and size of Tympanic Membrane perforation of Patients (n-36).

Characteristics	Frequency	Percentage (%)
*Age Groups (years)*		
± 20	7	19.4%
21-30	15	41.7%
31-40	13	36.1%
41-50	1	2.8%
*Gender*		
Male	21	58.3%
Female	15	41.7%
*Side of Ear*		
Right Side	22	61.1%
Left Side	12	33.3%
Both Side	2	5.6%
*Site of Perforation*		
Anterio-superior	8	22.2%
Anterio-inferior	19	52.8%
Posterio-superior	3	8.3%
Posterio-inferior	6	16.7%
*Size of perforation*		
*Medium perforation*	22	61.1%
*Large perforation*	10	27.8%
*Total perforation*	4	11.1%

**Table II:** Mean ± SD of pre-operative and post-operative air and bone conduction and air- bone gap on PTA and P- Value.

Items	Pre-Op (Mean± SD)	Post-Op (Mean± SD)	P-Value
Bone Conduction on PTA	14.72 ±6.43	14.72 ±6.43	.000
Air Conduction on PTA	49.72±11.08	18.27±5.06	<.05
Air-Bone Gap (ABG) on PTA	45.63±8.35	7.41±3.51	<.05

**Table III:** Comparison of changes in ABG, p-values and graft success rate of current study with other studies.

Author of study	No. of Patients	Year of study	Pre-Op ABG mean +/-SD(dB)	Post-OP ABG mean+/- SD (dB)	P-Value	Success rate
Subramania^2^	80	2017	22.4+/-6.14	14.8+/-10.2	<0.001	77%
Vaishali^3^	40	2017	22.6	13.6	<0.003	77.5%
Fattah^4^	40	2014	21+/-7	9.4+/-8.9	<0.007	80%
Gupta^5^	30	2015	33.27+/- 4.29	12.67+/-5.68	<0.001	90%
Khan^6^	28	2011	32.464 +/- 5.02	9.21+/3.28	<0.05	100 %
Kumar^7^	40	2014	22.6	13.6	<0.05	75%
Aydın^8^	60	2015	21.1+/-9.7	13.0+/-8.4	<0.05	96.7%
Sahan^9^	33	2014	24.5+/-7.2	12.8+/-5.6	<0.001	87.9%
Dhanapala^11^	100	2017	33.59 +/-3.8	26.87+/-3.87	<0.05	94%
Hasaballah^12^	40	2014	26.0 +/-± 4.4	13.8+/- 5	<0.0001	100 %
Pareek^13^	40	2017	25.45+/- 8.44	19.31+/-8.18	0.0014	100 %
Shrikrishna^14^	30	2015	22	10	<0.05	90%
Patil^15^	120	2010	21.54+/-6.36	10.97+/-3.68	0.0001	87.50%
Hodzic-Redzic^16^	243	2016	47.35+/-18.93	28.94+/-20.5	<0.0001	95.18%
Ocak^17^	179	2014	22.43+/-8.07	15.27+/-8.69	<0.001	86.5% %
Khan MM^18^	56	2013	39.89+/- 7.914	10.03+/-1.74	<0.05	100 %
Current Study	36	2017	45.63±8.35	7.41±3.51	<.05	94.4%

## DISCUSSION

In this study among 36 patients 21 (58.3%) were male and 15 (41.7%) were female with male: female ratio of 1.4:1, that is consistent with results of Subramania^2^ with 42 (52.55%) males, 38(47.5%) female and male: female ratio of 1.1:1, and Dhanapala with 57 (57%) males, 43(43%) female and male: female ratio of 1.3:1.^11^ However this study results regarding gender distribution differs from Vaishali, Hasaballah and Gupta having female predominance as female 55% male 45%, females 66.6% males 33.4% and females 70% males 30% respectively.^3^,^5^,^12^ In current study mean ± SD age was 27.14 ± 7.49 years, with age range 15 – 50 years and patients presented commonly in 3^rd^ and 4^th^ decade of life i.e. 15 (41.7%) and 13 (36.1%) respectively. Similarly in Pareek’s study mean ± SD age of the patient was 26.23 ± 12.46 years with age range of 10 to 60 years.^13^ Hasaballah observed that mean ± SD age was 24.9 ± 9.5 years with age range 15–51 years.^12^ Vaishali also reported that mean age of patients was 29.12 years (range, 15–45 years) and majority of patients (40%) were in 3^rd^ decade of life.^3^ In this study tympanic membrane perforation was commonly found on right side 22 (61.1%), predominantly involving anterio-inferior site 19 (52.8%) and medium sized perforation outnumbered 22 (61.1%), which is keeping with Dhanapala, who observed that right ear was involved in 55% patients while left ear was involved in 45% patients.^11^ Simply Shrikrishna found that medium sized perforation was commonest (56.7%), and Aydın also described that anterioinferior perforation was outnumbered (40%).^8^,^14^ Contrarily this study demographic values differ from Gupta’s finding where subtotal perforation was most common (83.3%).^5^ Patil also reported that subtotal perforation was common 49.17% followed by large central perforation in 51 cases (42.5%) differing from this study finding.^15^ In this study the mean pre-op air-conduction of 49.72 dB was significantly reduced to 18.27 dB. Similarly the pre-op mean air bone gap on PTA of 45.63 ± 8.35dB was also reduced to statistically significant level of 7.41±3.51 dB on post-op PTA, which is in conformity with result of Pareek, where mean ± SD preoperative air conduction of 43.21±7.17 dB was reduced to mean ± SD postoperative air conduction of 36.49 ± 6.60 dB, which was statistically significant (p-0.00004), while mean ± SD preoperative ABG of 25.45±8.44 dB was reduced to mean ± SD postoperative ABG of 19.31±8.18 dB, that was also statistically significant (p-0.0014).^13^ In current study hearing improvement after tympanoplasty was 77.8% and graft take up was 94.4%, which in accordance with Hodzic-Redzic’ study with hearing improvement of 60.24% and the graft success rate was 92.5%.^16^ The results of this study in terms of changes in ABG, p-values and graft success rate are compared to other studies tabulated in Table-III.

## CONCLUSION

Tragal perichondrial graft is an effective grafting material used for tympanoplasty due to its possessing qualities. There was statistically significant change in pre and post operative air-bone gap on pure tone audiometry, with statistically significant subjective hearing improvement. Graft success rate was 94.4% in this study, which signifies yield of tragal perichondrial graft tympanoplasty.

### Authors Contribution

**WFI:** Conceived, designed and prepared the manuscript.

**NSR:** Did statistical analysis & proof reading of manuscript.
